# Optimizing plant density to improve the soil microenvironment and enhance crop productivity in cotton/cumin intercropping systems

**DOI:** 10.3389/fpls.2025.1533211

**Published:** 2025-02-10

**Authors:** Humei Zhang, Liwen Tian, Xianzhe Hao, Nannan Li, Xiaojuan Shi, Feng Shi, Yu Tian, Wenbo Wang, Honghai Luo

**Affiliations:** ^1^ Key Laboratory of Oasis Eco-Agriculture, Xinjiang Production and Construction Group, Shihezi University, Shihezi, Xinjiang, China; ^2^ Western Agricultural Research Center, Chinese Academy of Agricultural Sciences, Changji, China; ^3^ Cotton Research Institute of Xinjiang Uygur Autonomous Region Academy of Agricultural Sciences, Urumqi, Xinjiang, China; ^4^ Key Laboratory of Northwest Oasis Water-Saving Agriculture, Ministry of Agriculture and Rural Affairs, Shihezi, China; ^5^ Agricultural Development Service Centre of the Seventh Division of the Xinjiang Production and Construction Corps, Kuitun, China

**Keywords:** cotton/cumin intercropping, density, soil environment, crop productivity, nonfilm cotton

## Abstract

**Introduction:**

Residual film pollution has become a key factor that affects the sustainable development of cotton, and intercropping may be an economical and environmentally friendly method to reduce the negative effects of nonmulched conditions on cotton growth. We hypothesized that optimizing the cotton/cumin intercropping density would improve the soil environment and increase crop productivity and resource utilization.

**Methods:**

Therefore, in this study, singlecropping cotton (CK) was used as the control, and three intercropping cumin seeding densities were used (plants ha^-1^: 5×10^5^, ID1; 8×10^5^, ID2; and 11×10^5^, ID3). Through a two-year field experiment, the effects of cotton-cumin intercropping on the soil moisture, temperature, salt, respiration rate, weed density, cotton yield formation and intercropping advantages were studied.

**Results and discussion:**

Compared with the CK treatment, the ID2 treatment decreased the water content in the 0–30 cm soil layer by 8.3%, increased the water consumption by 9.1%, increased the soil temperature by 0.5°C, and decreased the electrical conductivity of the 0–15 cm soil layer by 17.7%. Compared with the CK treatment, the ID1 treatment significantly decreased the soil respiration rate by 33.6%, and the weed density decreased in the following order: CK>ID1>ID2>ID3. During the nonsymbiotic period, compared with CK, ID2 increased the soil water content by 5.7%, increased the soil respiration rate by 17.7%, and decreased the electrical conductivity by 15.6%. Compared with those for CK and ID3, the seed yield for ID2 increased by 2.0% and 5.8%, respectively, and that for ID1 decreased by 1.6%. However, the land equivalent of the ID2 treatment was 4.3% greater than that for the ID1 treatment. Therefore, intercropping cumin at a density of 8×10^5^ plants ha^-1^ is beneficial for increasing surface coverage, significantly increasing crop water consumption, increasing surface temperature, reducing soil electrical conductivity and carbon emissions, and improving the crop yield and economic benefits. This model can be used as an agroecologically friendly and sustainable planting model.

## 1 Introduction

Cotton (*Gossypium hirsutum* L.) is a major cash crop and raw textile material worldwide (Tung et al., 2018). China accounts for 23.3% of the global cotton production, and the total cotton acreage and total production in Xinjiang account for 83.2% and 90.2%, respectively, of the totals in China (National Bureau of Statistics of China, 2022). The increase in cotton production in Xinjiang is due mainly to plastic film covering technology (Feng et al., 2017); currently, the amount of agricultural film produced in Xinjiang has reached 261000 tons (National Bureau of Statistics of China, 2021), and the recovery rate of residual film is less than two-thirds (Xue et al., 2021). The long-term accumulation of residual film can damage the soil particle structure, reduce soil permeability, increase the soil carbon dioxide concentration, inhibit root respiration, prevent crop absorption of water and nutrients, and reduce the crop yield (Wen et al., 2022; Li et al., 2023, 2024). In addition, the mixing of residual film during machine picking is an important cause of cotton quality degradation (Adeleke, 2023).

At present, the main methods used to prevent and control mulch pollution are biodegradable landfilm substitution and timely uncovering and recycling, but these methods are time-consuming, laborious and costly (Xue et al., 2021; Yang et al., 2023). Researchers have proposed cultivation models without film, which provide new methods to solve the problem of residual film pollution (Li et al., 2022a). However, the lack of a film has a strong negative effect on the increase in temperature, moisture conservation and salt grass suppression, which delays the fertility period and substantially reduces the crop yield (Yang B. et al., 2022; Li et al., 2023). In our previous research, we determined the optimal irrigation amount for high cotton yields under the conditions of nonfilm deep drip irrigation (Li et al., 2022a, 2022b). However, planting without film causes serious grass damage, and weeds highly compete with cotton for water and fertilizer resources, which reduces crop yields. Previous studies have shown that establishing appropriately spaced secondary crops (intercropping) that compete with weeds for resources is an effective nonchemical approach for curbing weed growth in nonfilm cotton fields (Weerarathne et al., 2017).

Intercropping has displayed significant advantages over monocropping in terms of the efficient utilization of light, temperature, water, and nutrient resources (Nyawade et al., 2019; Liang et al., 2020), reducing carbon emissions from fields (Bulson et al., 1997), suppressing weed growth and decreasing salt accumulation (Weerarathne et al., 2017; Liang and Shi, 2021). Intercropping also helps improve land utilization and crop productivity (Liang et al., 2020; Roohi et al., 2022) because of the spatial and temporal interspecific complementarity of intercropping systems (Raza et al., 2019). Dense planting is the key to increasing yield and efficiency in intercropping systems, and the planting density affects the competition and compensation efficiency of intercropping systems (Hauggaard-Nielsen et al., 2006). However, an excessive intercropping density can negatively impact the light-receiving structure of a population (Yang H. et al., 2022). Therefore, exploring reasonable planting densities in intercropping systems is conducive to optimizing the advantages of interspecific competition and compensatory benefits to increase crop yields.

Cumin (*Cuminum cyminum* L.) is the second most popular spice in the world (Sowbhagya, 2013). Cumin has notable intercropping advantages when it is planted in combination with cotton, maize, and other crops because of its high economic value, short growing period (65–70 days), high adaptability, and ability to be intercropped with few negative effects on light, water, and fertilizer patterns (Zhang et al., 2021). Although the cotton/cumin intercropping approach is widely used in plastic-film-mulched cotton fields in the southern region of Xinjiang, limited research has been conducted on this method in terms of the availability of soil water, temperature, salt, air, crop growth, and crop development. Thus, it is hypothesized that optimizing the cumin planting density in a cotton-and-cumin intercropping system with deep drip irrigation and without film can improve soil water availability, temperature and salt conditions, air quality and land productivity to compensate for the cotton yield and economic losses from not mulching.

The objectives of this study are (a) to analyze the effects of different cumin planting densities on the spatiotemporal distribution of soil water and temperature, (b) to explore the moderating effects of cumin density on factors such as soil salinity, respiration and weed growth, and (c) to identify the soil environmental factors that improve crop productivity in this system and to determine the optimum intercropping cumin density in this ecologically friendly cotton area. The results of this study provide a theoretical basis for the development of an efficient cultivation model for nonfilm cotton.

## 2 Materials and methods

### 2.1 Experimental site

The experiment was performed at the Experimental Station for Efficient Water Use in Agriculture of the Ministry of Agriculture and Rural Affairs in Shihezi, Xinjiang, China (45°38′N, 86°09′E; 430 m above sea level) during the 2022 and 2023 growing seasons.

**Figure 1:**
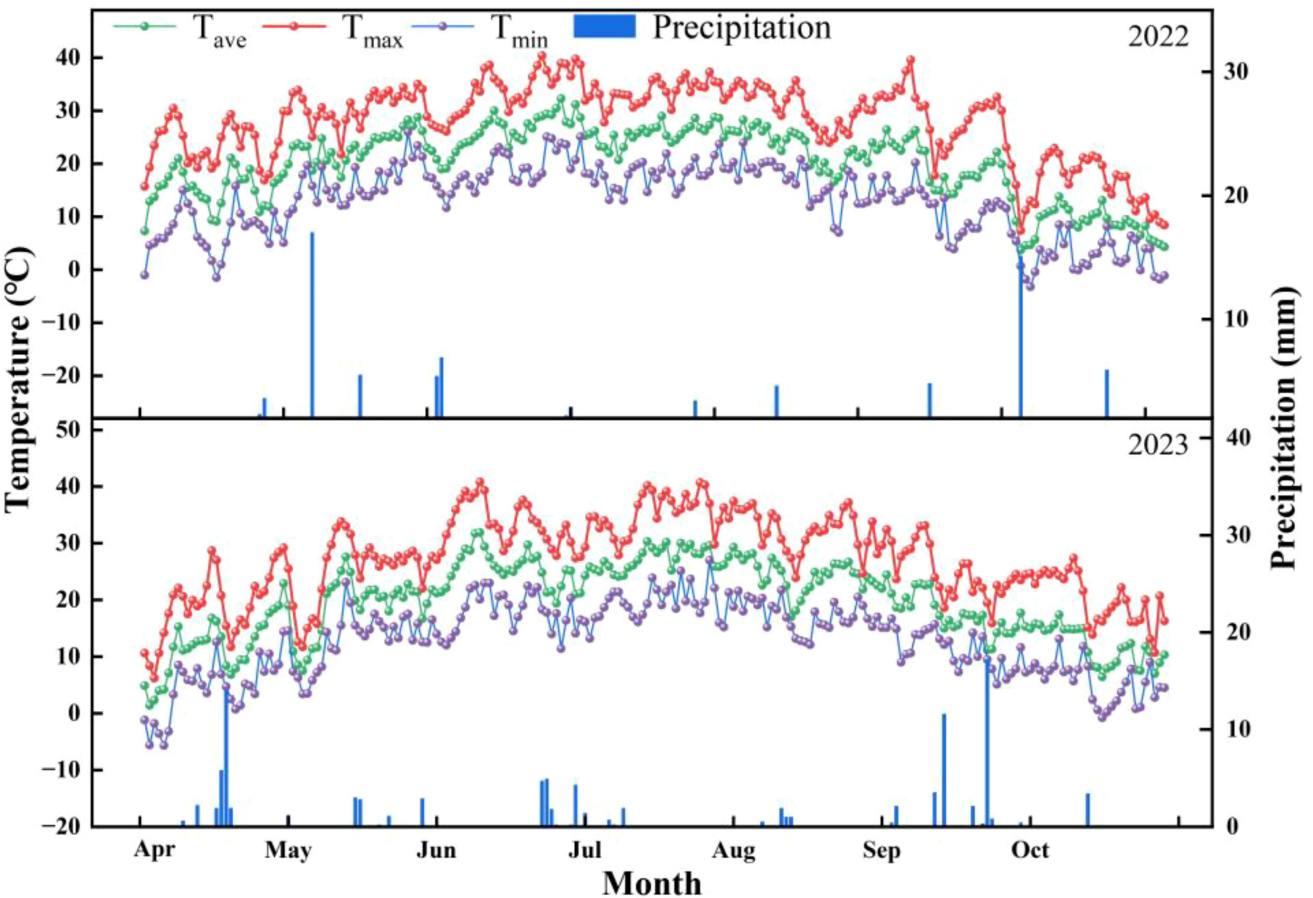
Monthly summary of the daily maximum/minimum temperature and precipitation during the cotton growing seasons in 2022 and 2023.

The cumulative rainfall during the cotton growing period (May 1 to October 18) in 2022 and 2023 was 157.0 and 156.3 mm, respectively, where the daily average maximum temperatures were 30.1 and 29.5°C, and the daily average minimum temperatures were 14.9 and 15.0°C, respectively (**Figure 1**). The basic soil properties in the 0–20 cm layer were as follows (**Table 1**). The previous crop was cotton. The tested cotton variety was the early-maturing upland cotton variety “Xinluzao 74” (growth period of 120 days) (Li et al., 2022a), and the cumin variety was “Cumin King 3”.

**Table 1:** Physical and chemical properties of the 0–20 cm soil layer.

Characteristic	Mean value
Soil bulk density (g cm^-3^)	1.30
pH	8.30
Electrical conductivity (μS cm^-1^)	510
Total nitrogen content (g kg^-1^)	1.30
Alkaline hydrolysis nitrogen (mg kg^-1^)	42.20
Available potassium (mg kg^-1^)	166.00
Available phosphorus (mg kg^-1^)	29.00
Organic matter (g kg^-1^)	23.00

### 2.2 Experimental design

**Figure 2:**
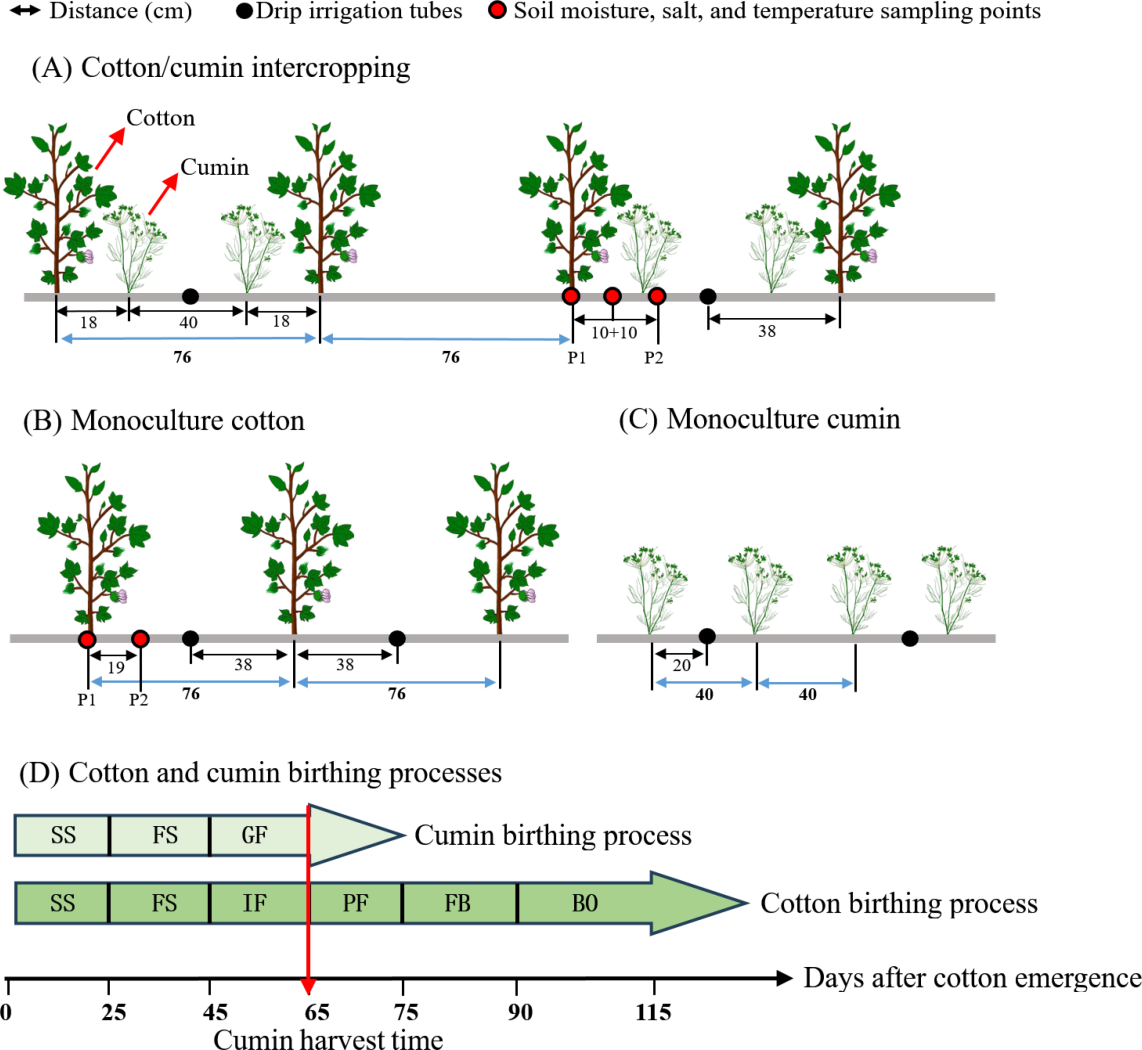
**(A–D)** Different planting patterns and the installation locations of the measurement equipment. P1: cotton root position; P2: intercropping row position. In D, the flowering process is described as follows: SS, seedling stage; FS, flowering stage; GF, grain filling stage; in the cotton germination process: SS, seedling stage; FS, full squaring stage; IF, initial flowering stage; PF, peak flowering stage; FB, full boll stage; BO, boll opening stage.

The experiment consisted of four treatments with a randomized complete block design and four replications. Three intercropping cumin seedling densities (5×10^5^ plants ha^-1^ (ID1), 8×10^5^ plants ha^-1^ (ID2) and 11×10^5^ plants ha^-1^ (ID3)) were selected, with monocropped cotton used as a control (CK). The cotton plants were spaced 5 cm apart, and the rows were 76 cm long. Two rows of cumin were planted between every two rows of cotton; the distance between the cumin rows and the neighboring cotton rows was 18 cm, whereas the distance between the two cumin rows was 40 cm (**Figure 2**). Each plot measured 45.6 m^2^ (7.6 m × 6 m).

After land levelling, special underground drip irrigation tubes were used (NETAFIM, Israel). The inner diameter of the dropper was 16 mm, the flow rate was 2.0 L h^-1^, and the distance between the emitters was 30 cm. Every two rows of cotton and every two rows of cumin shared a drip irrigation tube. The distance between the cotton rows and the drip irrigation tube was 38 cm, and the distance between the cumin rows and the drip irrigation tube was 20 cm (**Figure 2**). During the entire growth period, ordinary urea (46% N) and potassium dihydrogen phosphate (34% K_2_O and 52% P_2_O_5_) were applied with water droplets, resulting in 310 kg ha^-1^ N, 51 kg ha^-1^ K_2_O, and 78 kg ha^-1^ P_2_O_5_. Foliar spray fertilizer was applied as 0.3% potassium dihydrogen phosphate 1 week before cumin pollination and during the filling period. Cotton and cumin share the same water source. The plants were irrigated three times during the symbiotic period and six times during the nonsymbiotic period. The irrigation volume was 348 mm over the entire growth period (Li et al., 2021). Cotton and cumin were both sown on April 25, 2022, and April 26, 2023; the seedling emergence densities were 167000 plants ha^-1^ and 165000 plants ha^-1^, respectively. The cumin was harvested on July 10, 2022, and July 14, 2023, respectively. The other management practices used were identical to those used for conventional cultivation techniques (Feng et al., 2024).

### 2.3 Data collection

#### 2.3.1 Soil water content, soil water consumption and water use efficiency

The soil water content (SWC) was determined by drying samples collected with a stainless-steel auger during each growth period. **Figure 2** shows the sampling position, and the measurement depth ranged from 0–45 cm. The samples were stratified every 15 cm, mixed and placed in an aluminum box with three replicates. The samples were dried in an oven at 105°C until a constant weight was reached, after which the samples were weighed. The soil moisture content was calculated as follows:


(1) $$\text{SWC}=\frac{M_{2}-{\text{M}}_{1}}{M_{1}-M_{0}}\times 100\%$$

where M_2_ is the mass of the aluminum box with wet soil (g), M_1_ is the mass of the aluminum box with dry soil (g), and M_0_ is the mass of the aluminum box (g).


(2) $$\text{SC}=10\sum_{\text{i}=1}^{\text{n}}{\text{ρ}}_{\text{i}}{\text{H}}_{\text{i}}({\text{SWC}}_{\text{i}1}-{\text{SWC}}_{\text{i}2})+\text{I}+\text{P}$$

The soil water consumption (SC, mm) of crops during different growth stages can be determined via the water balance equation. In the equation, i is the number of soil layers, n is the total number of soil layers, ρ_i_ is the soil bulk weight of layer i (g cm^-3^), H_i_ is the thickness of layer i (cm), SWC_i1_ is the water content of layer i at the beginning of the growing period (%), SWC_i2_ is the water content of layer i at the end of the growing period (%), i is the amount of irrigation water during the reproductive period (mm), P is the amount of rainfall during the reproductive period (mm), and precipitation during the entire reproductive period can be neglected (Li et al., 2022a).

The water use efficiency (WUE) was calculated as follows:


(3) $$\text{WUE}=\frac{\text{Y}}{\text{S}{\text{C}}_{\text{t}}}$$

where Y is the total crop yield (kg ha^-1^; formula 3) and SC_t_ is the total crop water consumption (mm).

#### 2.3.2 Soil temperature

A right-angle ground thermometer (Great Wall Instrumentation Factory, China) was used. The temperature was measured in the range of -20-50°C, and the accuracy was 0.5°C. The temperatures in the 5-, 15-, and 25-cm soil layers were measured during each growth period, and **Figure 1** shows the locations of the measurements. The average temperatures in the morning (8:00), at noon (14:00) and in the evening (20:00) were selected as the daily average soil temperatures. Daily changes in temperature in the 5-cm soil layer were measured at the initial flowering stage (symbiotic period) and the full boll stage (nonsymbiotic period) of cotton. The temperatures were recorded every 2 h from 8:00 am to 20:00 pm.

#### 2.3.3 Soil electrical conductivity

Soil salinity sampling and moisture sampling were conducted in the same batch. Samples from the 0–15, 15–30, and 30–45 cm soil layers were placed in self-sealing bags, naturally air-dried, and ground to pass through a sieve with a 1 mm mesh size. The conductivity of the soil (EC, soil/water =1:5, uS cm^-1^) was determined via a conductivity meter (DS-307, China).

#### 2.3.4 Soil respiration rate

Soil respiration was measured in the field via an automated soil carbon flux measurement system (LI-8100A, LI-COR, USA) during each growth period. A sunny day with little wind or clouds was selected, and each measurement was from 10:00–13:00 Beijing time. Changes in the soil respiration rate were measured at the initial flowering stage (symbiotic period) and full boll stage (nonsymbiotic period) every 2 hours from 9:00–19:00 pm. To avoid errors in the test system caused by differences in measurement times, a cyclic measurement method was adopted for different treatments.

To reduce interference with the soil surface, the soil respiration chamber was placed on a measuring base, which was a polyvinyl chloride (PVC) ring with a height of 11 cm and a diameter of 20 cm. The measuring base was embedded in the soil and exposed to the soil surface for 2 cm. The measurement site was located in the middle of the cotton and cumin fields. The day before each measurement began, all fallen material, live plants and fauna were removed from the soil surface at the measurement site, and the burial position of the measurement site remained unchanged throughout the measurement process (Qin et al., 2013).

#### 2.3.5 Weed density and strain control effects

In accordance with the methods of Dang et al. (2017), weed populations were surveyed in 2022 at the seedling and full squaring stages of cotton and in 2023 at the seedling, full squaring, and initial flowering stages of cotton. Three 50 cm × 50 cm uniformly growing subplots were randomly selected in each plot to investigate the number of weeds in the sample plot and calculate the weed density.


(4) $$WD=\frac{N}{S}$$

where WD is the weed density (plant m^-2^), N is the number of weed plants (plant), and S is the survey area (m^2^).

After the number of weeds in the sample plot was investigated, all the weeds in the sample plot were removed, washed and weighed fresh. The corresponding formula is as follows:

strain control effect (%)= (number of weeds in the control group - number of weeds in the treatment group)/number of weeds in the control group×100%.

Fresh weight control effect (%)= (weed fresh weight in the control group - weed fresh weight in the treatment group)/weed fresh weight in the control group×100%.

#### 2.3.6 Yield, land equivalent ratio and aggressivity

Seed cotton was manually picked in a unit area (2 m × 1.52 m) during the cotton harvest (October 13, 2022, and October 16, 2023), and the numbers of plants and bolls (diameter >3 cm) per unit area (3 ×1.52 m) were determined. Ten cotton bolls were randomly collected from each plot to measure the weight of each boll and calculate the theoretical yield. In the cumin harvest season, a unit area (1.05 m × 2 m) sample subplot was selected in each plot; then, a sample from each subplot was evenly uprooted, naturally air-dried, and weighed. Finally, the grain yield was calculated.

The densities of monocropped and intercropped cumin were identical at identical planting densities and net sown areas. Monocultures cumin was used to calculate indicators related to interspecific competition.

The land equivalent ratio (LER) was calculated as follows (Mead and Willey, 1980):


(5) $$\text{LER}=(\frac{{\text{Y}}_{\text{ic}}}{{\text{Y}}_{\text{mc}}})+(\frac{{\text{Y}}_{\text{if}}}{{\text{Y}}_{\text{mf}}})$$

where Y_ic_ and Y_if_ are the yields of intercropped cotton and cumin, respectively, and Y_mc_ and Y_mf_ are the yields of monocropped cotton and cumin, respectively. An intercropping advantage occurs when LER>1.0, and an intercropping disadvantage occurs when LER<1.0.

The aggressivity of cotton relative to cumin was calculated as follows:


(6) $$\text{Am}=(\frac{{\text{Y}}_{\text{ic}}}{{\text{Y}}_{\text{mc}}})-(\frac{{\text{Y}}_{\text{if}}}{{\text{Y}}_{\text{mf}}})$$

When Am=0, there is no competition between the two crops; when Am>0, cotton has a greater competitive advantage than does cumin.

### 2.4 Statistical analysis

The data were processed via Microsoft Excel 2021 software. Statistical analyses were performed via SPSS 19.0 software (SPSS, Chicago, USA). Significance tests were performed via Duncan’s method (P<0.05). Images were plotted with Origin 2023b and SigmaPlot 12.5 software.

## 3 Results

### 3.1 Soil water content and crop water consumption

**Figure 3:**
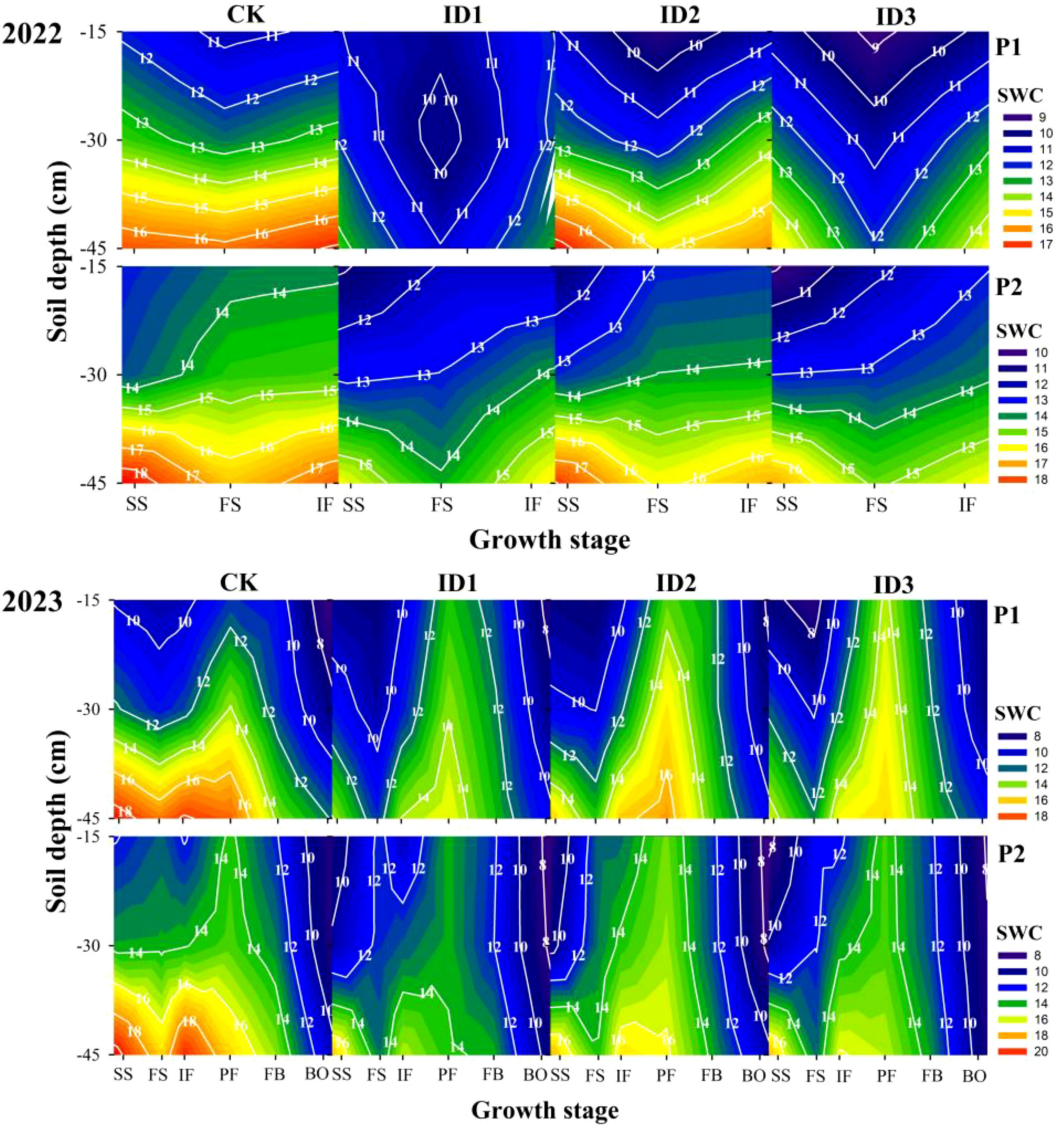
Dynamic changes in soil water content in the 0-45-cm soil layer under different treatments. Cotton/cumin intercropping symbiotic period: seeding stage (SS), full squaring stage (FS), peak flowering stage (IF); cotton/cumin intercropping nonsymbiotic period: peak flowering stage (PF), full boll stage (FB), boll opening stage (BO). P1: cotton root position; P2: intercropping row position.

The cotton/cumin intercropping system significantly affected the horizontal and vertical changes in SWC, where the SWC decreased in the following order: monocropping > intercropping and P1 < P2 (**Figure 3**). The differences among the treatments were not significant in the 0–15 cm soil layer during the cotton/cumin intercropping symbiotic period. At point P1 in 2022, compared with the CK treatment, the ID1 and ID3 treatments significantly (P<0.05) decreased the SWC in the 15-45 cm soil layer at the initial flowering stage by 18.7% and 11.5%, respectively; however, there was no significant (P>0.05) difference between the CK and ID2 treatments. The ID2 treatment displayed the greatest soil moisture retention at point P2. In 2023, the SWC at the full squaring stage significantly decreased in all the treatments; in the 30-45 cm soil layer from the seedling stage to the full squaring stage, all the treatments significantly (P<0.05) reduced the SWC by 19.6-51.3% compared with that in CK. During the nonsymbiotic period, ID1, ID2, and ID3 had 11.5%, 17.5%, and 18.7% (P<0.05) higher average SWC, respectively, in the 0–15 cm layer than did CK, and there was no significant (P>0.05) difference among the treatments in the 15–45 cm soil layer.

During the cotton/cumin intercropping symbiotic period, the soil water consumption (SC) in the 0–45-cm soil layer followed the order of intercropping > monocropping; the SC in the 0–15-cm and 30–45-cm layers increased and decreased, respectively, with increasing intercropping cumin density (**Table 2**). In 2022, the SC in the 15-45 cm layer was significantly greater in each intercropping treatment than in the monoculture, but there was no significant difference among the other soil layers. During the nonsymbiotic period, no treatment had a significant effect on the SC, which was greater in the monoculture treatment than in the intercropping treatment at P2. Thus, over the entire growth period, cotton/cumin intercropping reduced the SC in the 0–15 cm layer but had no significant effect in the other soil layers. The population density during the symbiotic period had a significant (P<0.01) effect on the SC in the 0–30 cm layer at P2 in all cases, as did the planting year in the 0–45 cm layer at P2. The treatment × year interaction significantly (P<0.05) affected the SC in the 15–30 cm layer at P2.

**Table 2:** Soil water consumption (mm) in the 0–45 cm layer under the different treatments.

Growth period	Treatment	P1			P2		
		0-15 cm	15-30 cm	30-45 cm	0-15 cm	15-30 cm	30-45 cm
2022	CK	63.58a	70.02b	79.64b	62.56b	66.19a	74.33b
Symbiosis period	ID1	63.93a	87.39a	87.96a	63.78ab	68.03a	89.06a
	ID2	67.67a	86.89a	81.07ab	65.52ab	69.18a	84.42a
	ID3	67.92a	84.24a	83.02ab	66.97a	67.26a	84.97a
2023	CK	63.81b	73.82a	91.57a	65.11b	65.44b	65.19a
Symbiosis period	ID1	64.71ab	77.03a	103.99a	72.13a	77.30a	73.92a
	ID2	65.44ab	77.15a	102.11a	72.86a	83.26a	70.43a
	ID3	70.44a	79.58a	99.41a	73.20a	77.14a	67.08a
Nonsymbiosis period	CK	283.45a	313.80a	331.02a	286.95a	316.33a	307.90a
	ID1	286.56a	310.62a	331.90a	288.13a	308.86a	289.52a
	ID2	289.10a	322.33a	341.64a	287.85a	316.71a	299.68a
	ID3	289.21a	319.97a	331.66a	288.28a	310.46a	296.65a
Entire growth period	CK	347.26b	387.62a	422.59a	352.06ab	381.77a	373.09a
	ID1	351.27ab	387.65a	435.89a	360.25a	386.16a	363.44a
	ID2	354.54ab	393.48a	443.75a	360.71a	397.96a	366.76a
	ID3	359.65a	399.56a	431.07a	365.47a	389.60a	367.08a
P value during symbiosis period							
Treatment		ns	ns	ns	**	**	ns
Year		ns	ns	ns	**	**	**
Treatment × year		ns	ns	ns	ns	*	ns

Intercropping symbiotic period: seedling stage (SS), full squaring stage (FS), peak flowering stage (IF); Intercropping nonsymbiotic period: peak flowering stage (PF), full boll stage (FB), boll opening stage (BO). P1: cotton root position, P2: intercropping row position. Means within a column followed by a different letter are significantly different (P < 0.05) according to Duncan’s multiple range test. *p<0.05, **p<0.01, ns: *p*>0.05.

### 3.2 Soil temperature

**Figure 4:**
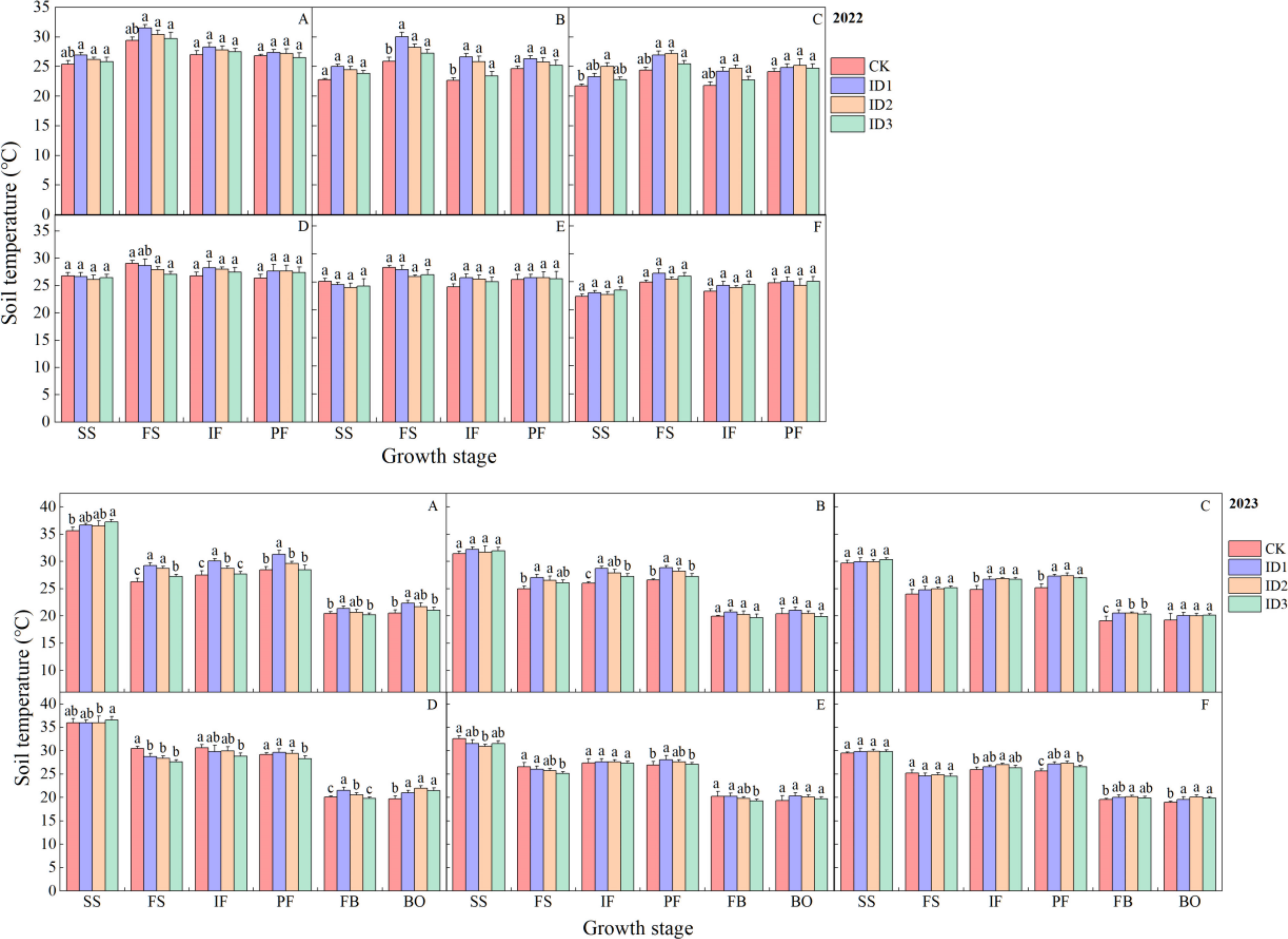
Dynamic changes in soil temperature at 0–25 cm depth under different treatments. Intercropping symbiotic period: seedling stage (SS), full squaring stage (FS), peak flowering stage (IF); Intercropping nonsymbiotic period: peak flowering stage (PF), full boll stage (FB), boll opening stage (BO). P1: cotton root position, P2: intercropping row position. **(A-C)** represent the 5, 15, and 25 cm soil layers in the cotton root position (P1), and **(D–F)** represent the 5, 15, and 25 cm soil layers in the intercropping row position (P2), respectively. Different lowercase letters indicate significant differences between treatments (p<0.05).

The effect of the cotton/cumin intercropping system on the soil temperature (ST) varied with the planting density. The ranking of the STs at P1 during the entire growth period was monocropping < intercropping (**Figure 4**). ST peaked at the full squaring stage in 2022. During the entire growth stage, the STs at point P1 exhibited the following ranking: ID1 > ID2 > ID3 > CK, where ID1, ID2, and ID3 had 8.4%, 7.2%, and 2.8% higher STs than CK, respectively. At P2, the 15-cm layer in the SS-FS intercrop was 0.4–1.7°C cooler than that in the monocrop, and the P2 point was not significantly different among the treatments at different growth stages. In 2023, in the 5–15 cm soil layer at the P1 point, compared with that in the CK treatment, the soil temperature increased by 2.5–11.2% and 0.7–9.3% in the symbiotic periods ID1 and ID2, respectively. The soil temperature was significantly (P<0.05) greater at FS and IF than CK. The soil temperature was significantly (P < 0.05) lower in the 5-cm soil layer at point P2 in the intercropping treatment than in the monocropping treatment and was lowest in the ID3 treatment during the other periods.

**Figure 5:**
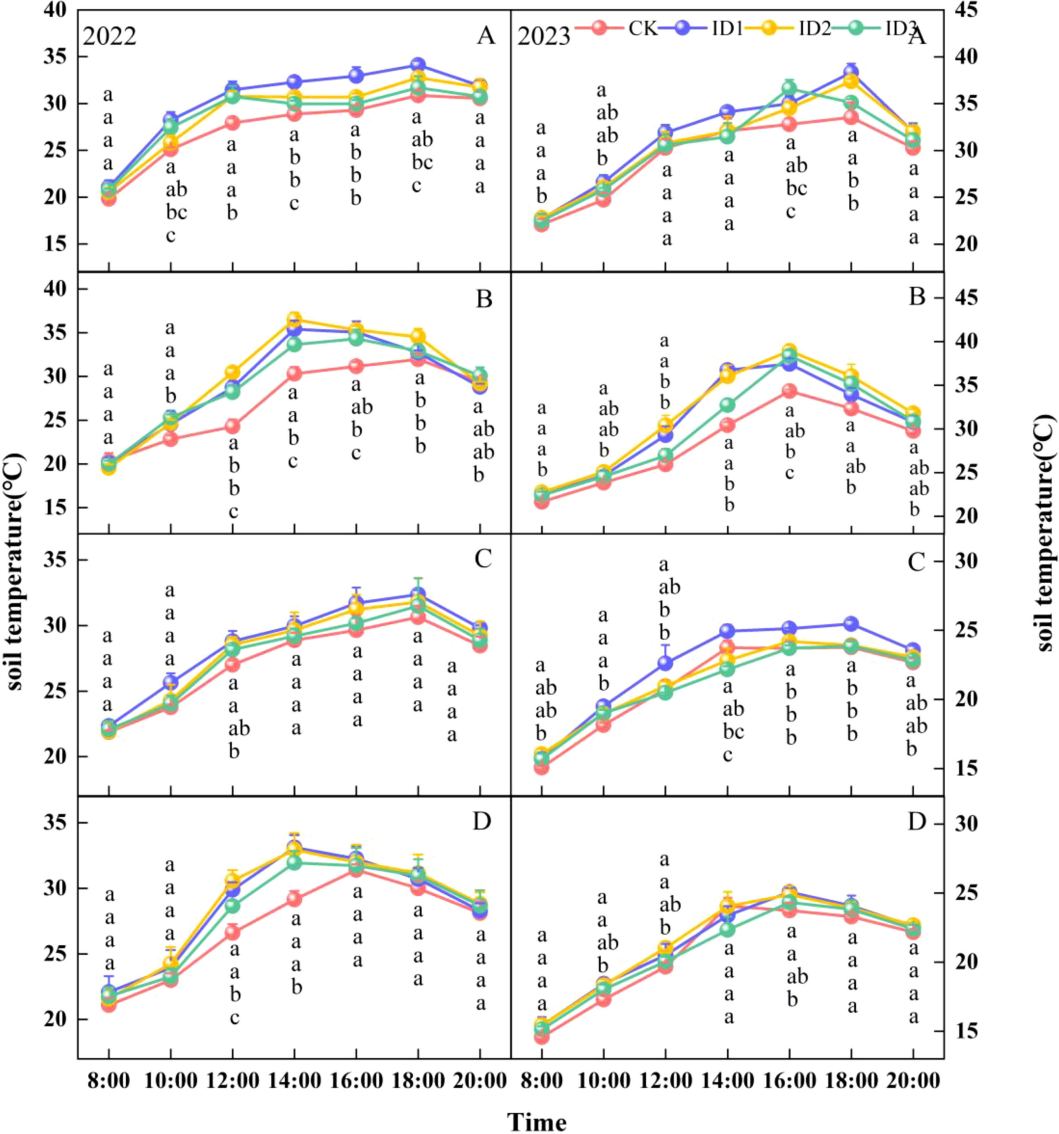
Daily variation in soil temperature in the 5-cm soil layer at the initial flowering (IF) and full-boll (FB) stages under different treatments. **(A, B)** represent the cotton root position (IF-P1) and intercropping row position (IF-P2) at initial flowering; **(C, D)** represent the cotton root position (FB-P1) and intercropping row position (FB-P2) at the full boll, respectively. Different lowercase letters indicate significant differences between treatments (p<0.05).

The soil temperature first increased but subsequently decreased over time (**Figure 5**). The STs at P1 and P2 reached their peaks at 18:00 and 14:00, respectively. At 10:00–18:00, compared with those of the CK and ID3 treatments, the average ST of ID1 was significantly greater by 3.4–1.8°C and 3.1–0.4°C, respectively (**Figures 5A, B**); at FB-P2, the ST in ID1 at 12:00 was 3.3 and 1.2°C greater than those in the ID3 and CK treatments, respectively (**Figure 5D**). There were no significant differences among the treatments at other times. In 2023, the ID3 treatment had the highest soil temperature at 16:00 under FB-P1 conditions, with significant (P<0.05) increases of 3.8 and 2.1°C compared with those of CK and ID2, respectively. However, the ST at P2 in ID2 significantly (P<0.05) differed from that in the CK treatment but did not significantly differ from that in the ID1 treatment (except at 14:00) (**Figure 5A**); at FB-the P1, the temperature in ID1 was 5.8–7.7% higher than those in the other treatments from 12:00–20:00 (**Figure 5C**).

### 3.3 Soil electrical conductivity

**Figure 6:**
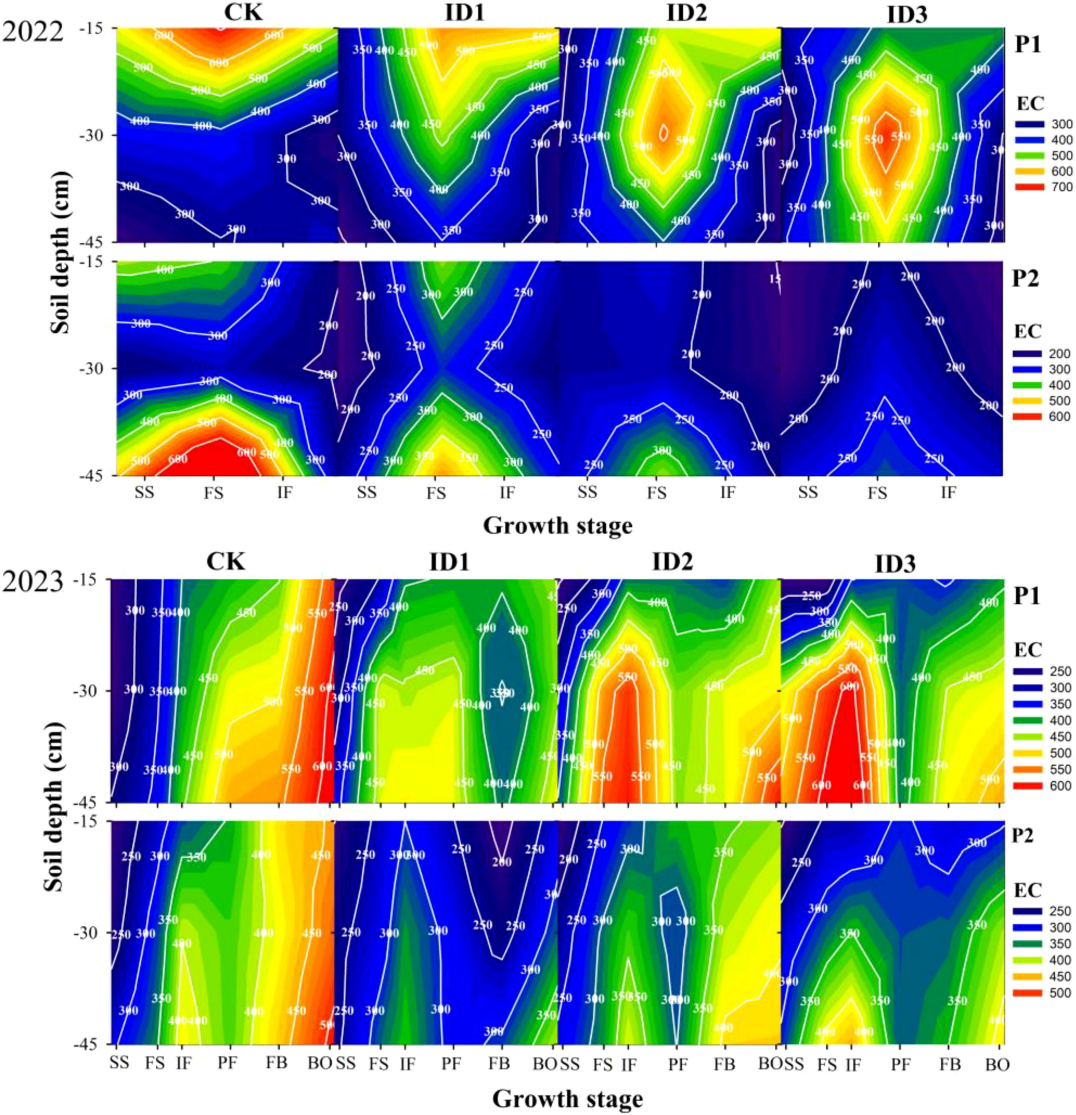
Dynamic changes in soil electrical conductivity in the 0–45 cm soil layer under different treatments. Intercropping symbiotic period: seedling stage (SS), full squaring stage (FS), peak flowering stage (IF); Intercropping nonsymbiotic period: peak flowering stage (PF), full boll stage (FB), boll opening stage (BO); P1: cotton root position, P2: intercropping row position.

The soil electrical conductivity (EC) tended to gradually increase with increasing soil depth (except for 2022-P1) and longer growth period. In the horizontal direction, EC displayed the relation of P1 > P2 (**Figure 6**). At points P1 and P2 in 2023, the 0-15-cm soil layer had 6.4-37.0% and 7.3-19.1% lower EC than did the 15-30-cm layer and 12.7-41.7% and 13.8-28.7% higher EC than did the 30-45-cm layer, respectively. During the symbiotic period, in the 0–15 cm soil layer, the EC of each treatment decreased in the order of CK > ID1 > ID2 > ID3, and the ID2 and ID3 treatments had 6.0–41.6% and 8.2–20.4% lower EC than the CK treatment did. However, in the 30–45 cm soil layer, at point P1, the EC displayed the order of ID3 > ID2 > ID1 > CK. During the nonsymbiotic period, at points P1 and P2, the CK and ID1 treatments had the highest and lowest ECs in the 0-45 cm soil layer, respectively. Compared with CK, the ID1, ID2 and ID3 treatments decreased the EC by 13.0–42.3%, 12.0–19.6% and 18.1–28.6%, respectively.

### 3.4 Soil respiration rate

**Figure 7:**
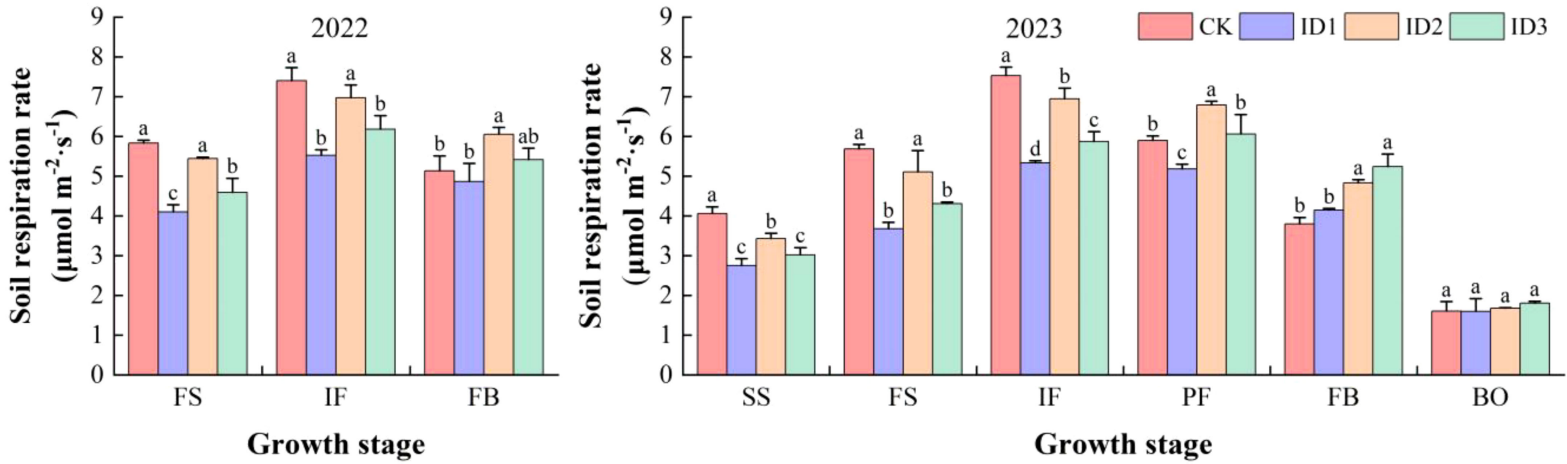
Dynamic changes in the soil respiration rate at different growth stages under different treatments. Intercropping symbiotic period: seedling stage (SS), full squaring stage (FS), peak flowering stage (IF); Intercropping nonsymbiotic period: peak flowering stage (PF), full boll stage (FB), boll opening stage (BO). Different lowercase letters indicate significant differences between treatments (p<0.05).

The soil respiration rate (SR) in the treatments in the cotton/cumin intercropping system peaked at the IF (**Figure 7**). During the cotton/cumin intercropping symbiotic period, the CK and ID1 treatments had the highest and lowest SRs, respectively. Compared with those for the CK treatment, the SRs of the ID2 and ID3 treatments were significantly (P<0.05) lower (by 27.6% and 18.9% and by 31.2% and 22.7%, respectively). Compared with the CK treatment, the ID2 and ID3 treatments significantly (P<0.05) increased the SR by 17.9% and 5.5%, respectively, during FB in 2022, whereas there was no significant difference between the ID1 and CK treatments. In the PF to FB stages in 2023, ID2 exhibited 14.9% and 19.4% higher SRs than did CK and ID1, respectively, but there was no significant difference between ID2 and ID3, and there was no significant treatment difference at BO.

**Figure 8:**
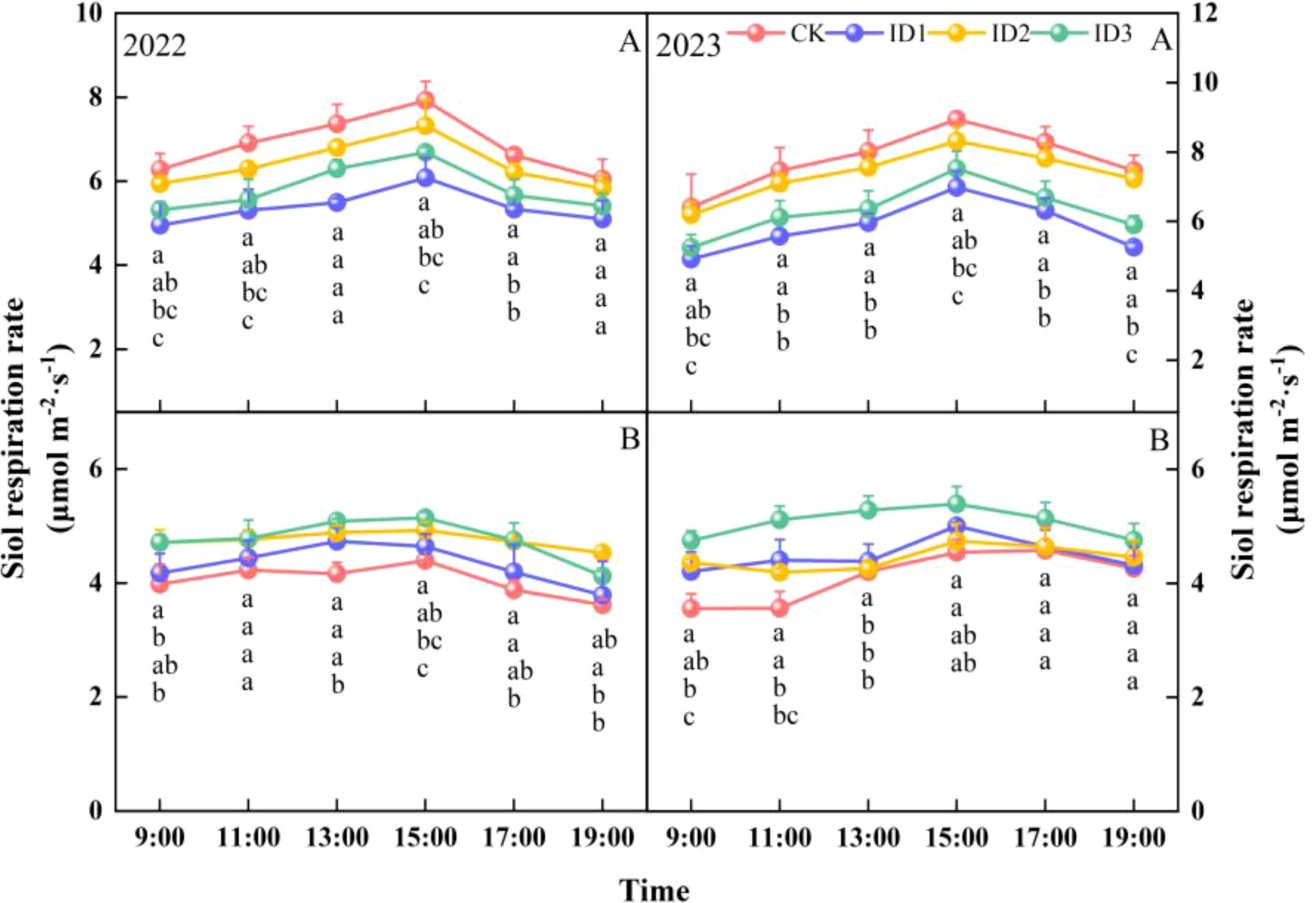
Daily variation in the soil respiration rate at the initial flowering **(A**, IF) and full-boll **B**, FB) stages under different treatments. Different lowercase letters indicate significant differences between treatments (p<0.05).

At IF, the SR first increased, peaked at 15:00, and subsequently decreased over time (**Figure 8A**). In 2022, the average daily SRs of CK, ID1, ID2, and ID3 were 6.9, 5.4, and 6.4, respectively; in 2023, the average daily SRs of CK, ID1, ID2, and ID3 were 5.8 and 7.5, 5.3, 7.2, and 5.9 μmol m^-2^ s^-1^, respectively. Compared with those in CK, the daily average SRs in ID3 and ID1 were significantly (P<0.05) lower by 27.6% and 17.8% in 2022 and 33.1% and 23.1% lower in 2023, respectively, whereas there was no significant difference between CK and ID2. ID3 and CK presented the highest and lowest SRs during FB, respectively (**Figure 8B**). Compared with those for CK and ID1, the mean daily SR for ID3 significantly (*P* < 0.05) increased by 17.9% and 10.2% in 2022, respectively. During the period of 09:00–15:00 in 2023, ID3 significantly (*P* < 0.05) increased the SR by 29.5% and 17% compared with those of CK and ID2, respectively, and there was no difference among the treatments after 15:00.

### 3.5 Weed suppression effect

As shown in **Table 3**, in 2022, compared with the CK treatment, ID1, ID2 and ID3 significantly (*P* < 0.05) decreased the weed density by 25.9%, 24.1% and 36.2%, respectively, during the seedling stage, and there was no significant difference among the treatments in the intercropping system. Compared with the CK treatment, ID2 and ID3 significantly (*P* < 0.05) decreased the weed density by 19.4% and 50.0%, respectively, in the full squaring stage, and there was no significant difference between ID1 and CK. In 2023, during the seedling and initial flowering stages, ID2 and ID3 significantly (*P* < 0.05) decreased the weed density by 46.4% and 60.7% and by 50.0% and 57.1%, respectively, compared with CK, whereas ID1 did not significantly differ from CK. In the full squaring stage, ID2 and ID3 had 25.6% and 66.9% stronger fresh weight control effects than did ID1, respectively; in the initial flowering stage, these (*P* < 0.05) differences were significant and reached 60.3% and 80.3%, respectively.

**Table 3:** Inhibitory effects of different treatments on field weeds during the symbiotic period.

Year	Survey indicators	Treatments	SS	FS	IF
2022	Weed density (plant m^-2^)	CK	29.00a	18.00a	—
		ID1	21.50b	17.00a	—
		ID2	22.00b	14.50b	—
		ID3	18.50b	9.00c	—
2023	Weed density (plant m^-2^)	CK	22.40a	14.40a	11.20a
		ID1	20.00a	13.60a	7.20ab
		ID2	12.00b	12.80a	5.60b
		ID3	8.80b	11.20a	4.80b
	Strain control effect (%)	ID1	10.71b	5.56a	36.71ab
		ID2	46.43a	11.11a	50.00a
		ID3	60.71a	22.22a	57.14a
	Fresh weight control effect (%)	ID1	67.25b	47.24c	45.48b
		ID2	83.76ab	59.50b	72.94a
		ID3	89.56a	78. 86a	81.98a

Intercropping symbiotic period: seedling stage (SS), full squaring stage (FS), peak flowering stage (IF); Means within a column followed by a different letter are significantly different (P < 0.05) according to Duncan’s multiple range test.

### 3.6 Crop yield and land equivalent ratio

Cotton/cumin intercropping had effects on the cotton yield and the total number of cotton bolls but had no significant effect on boll weight (except in 2022). In 2022 and 2023, compared with those in the CK treatment, the seed cotton yields in the ID1 and ID2 treatments increased by 1.9% and 1.1% and 5.4% and 2.1%, respectively, whereas the seed cotton yields in the ID3 treatment decreased by 6.3% and 0.7%, respectively. In 2022, ID1 had the greatest total boll number, and ID2 had the greatest boll weight, which significantly (*P* < 0.05) increased by 4.8% and 3.8% compared with those in the CK treatment, respectively, although there was no difference from those in the other treatments. In 2023, the seed cotton yield and total number of bolls in the ID1 treatment significantly (*P* < 0.05) increased by 6.2% and 3.8%, respectively, compared with those in the ID3 treatment. The yields of intercropped cumin were ID2>ID3>ID1, and ID2 displayed an 11.7–66.6% greater cumin yield than the other treatments did. Water utilization was greater under intercropping than under monocropping.

The LER ranged from 1.59-1.75, and the Am ranged from 0.26-0.41. The population density significantly (*P* < 0.05) affected the seed yield and intercropped cumin yield. The planting year had a significant effect on the intercropped cumin yield. However, the treatment × year interaction had no significant effect on the results.

### 3.7 Correlation analysis

**Figure 9:**
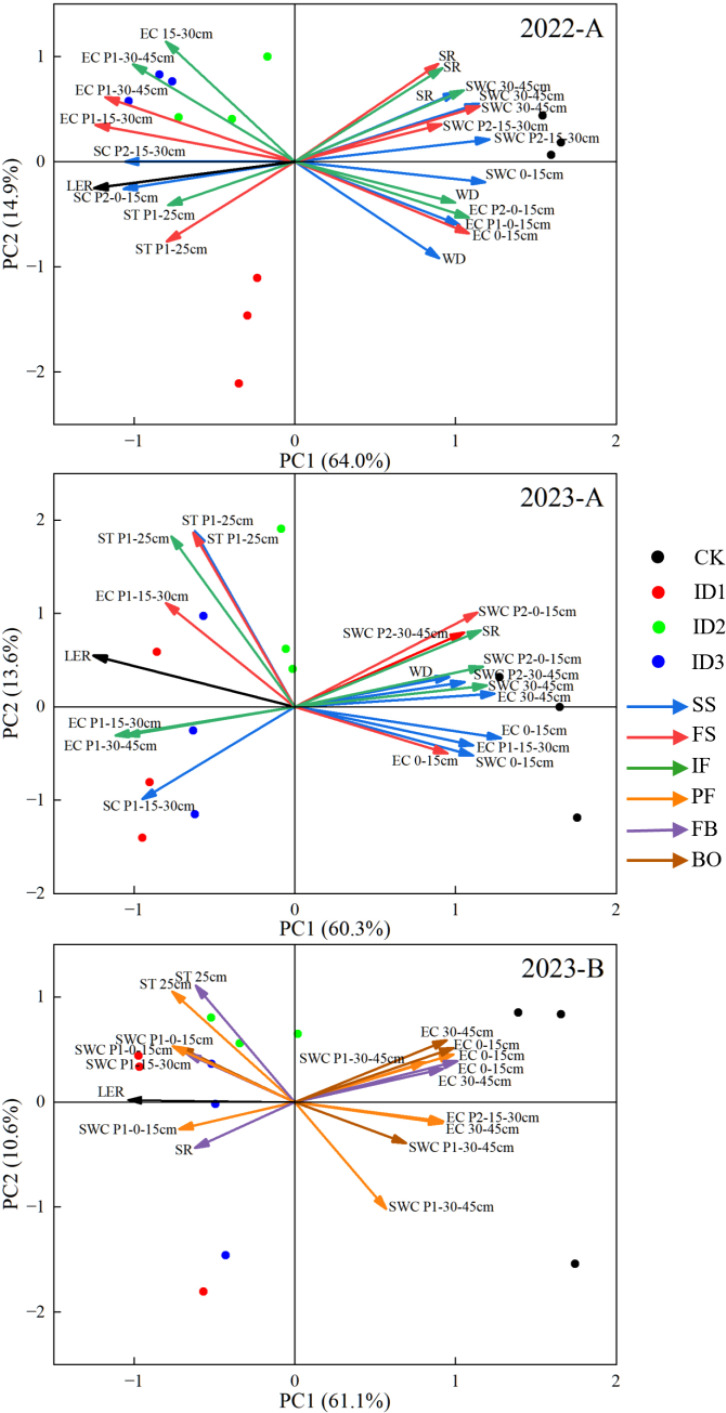
Principal component analysis of the soil microenvironment and yield. **(A)** Cotton/cumin intercropping symbiotic period; **(B)** Cotton/cumin intercropping nonsymbiotic period. LER is the land equivalent ratio, SWC is the soil water content, EC is the electrical conductivity, ST is the soil temperature, SR is the soil respiration rate, and SC is the soil water consumption.

During the cotton/cumin intercropping symbiotic period, the principal components on the first axis accounted for 64.0% and 60.3% of the total variation in 2022 and 2023, respectively (**Figure 9**). The land equivalent ratio was positively correlated with the ST in the 25-cm layer at point P1 and with the SC and EC in the 15-30-cm layer. However, the land equivalent ratio was negatively correlated with the SWC in the 15–45 cm layer, SR in the full squaring stage, WD at the seedling stage in 2022 and initial flowering stage in 2023, and EC in the 0–15 cm layer in the full squaring stage (**Figure 9A**). This finding indicates that the two crops strongly competed for water during the symbiotic period, the crop roots fully absorbed water from the 15-45 cm soil layer, and surface cover increased the temperature and promoted yield formation. In 2023, the percentage variances of the principal components on the first and second axes in the nonsymbiotic period were 61.1% and 10.6%, respectively (**Figure 9B**). The CK treatment significantly differed from the other treatments, where the PC1 land equivalent ratio had the largest loading value of 0.30, followed by P1-25 cm ST at FB. The LER was positively correlated with ST at 25 cm at the peak flowering–full boll stage, SWC in the 0–15-cm layer and SR at the full boll stage. The LER was negatively correlated with EC at 0–45 cm and SWC at 30–45 cm at P1.

## 4 Discussion

### 4.1 Effects of cotton/cumin intercropping on the spatial and temporal distributions of soil water and temperature

Moisture plays a key role in crop growth and development (Bai et al., 2023), and the planting density can alter crop competition for water and nutrients (Zheng et al., 2018; Da Silva et al., 2023). In this study, during the cotton/cumin intercropping symbiotic period, the soil water content in the intercropping system was 2.4–31.2% lower than that in the monoculture system (**Figure 3**), which is different from the results of previous studies of cotton/wheat intercropping (Wu et al., 2022b). This difference may be due to the vigorous growth of cumin in the early stage (Zhang et al., 2021), during which soil water consumption is high. The water consumption in the intercropping system was 0.6–27.2% greater than that in the monoculture system, and the 0–15 cm soil layer displayed the highest water consumption (**Table 2**), likely because the cumin roots were distributed mainly in this soil layer, which accelerated water absorption and consumption. In addition, ID1 had the highest soil water consumption in the 15-45 cm layer, likely because the lower crop cover increased soil evapotranspiration (Chai et al., 2011; Da Silva et al., 2023). ID2 exhibited the greatest SWC in the 0–30 cm layer during the nonsymbiotic period (**Figure 3**). These findings indicate that an appropriate cotton/cumin intercropping density can enhance soil moisture utilization in layers and alleviate problems such as ineffective evaporation and loss of water resources in the early stage of growth for nonfilmed cotton plants.

Soil temperature is a comprehensive indicator of the thermal status of the soil, which acts directly or indirectly on the various processes of crop growth and development (Jacobs et al., 2011). Different row spacing configurations and planting densities lead to differences in soil temperature (Moreira et al., 2015), and the soil temperature of mulched soil is 2–4°C greater than that of nonfilm mulched soil (Braunack et al., 2015). This study revealed that in the 5–25 cm layer during the entire growth period, the soil temperature in ID1 and ID2 was 1.0–2.0°C greater than that in CK (**Figure 4**), whereas previous results showed that intercropping reduced the soil temperature (Nyawade et al., 2019; Ai et al., 2021). First, this difference could be due to slow surface warming and the large temperature difference between day and night for bare ground plantings. The difference in temperature between ID1 and ID2 compared with CK at 8:00 and 20:0 during IF was 0.4–1.3°C (**Figure 5**). Second, the soil moisture decreased and the soil temperature increased because the intercropping treatments consumed more water than monocropping did (Onwuka and Mang, 2018). Thus, these findings indicate that cotton/cumin intercropping provides a general warming benefit throughout the entire growth period, which reduces the risk associated with low temperatures during early planting. In addition, intercropping systems have greater soil temperature stability than monocropping systems do (Ai et al., 2021), and intercropping mitigates the drastic interseasonal and diurnal changes in soil temperature caused by the lack of plastic film mulching (Wang et al., 2022b; Li et al., 2023), which increases the stability of the soil temperature.

### 4.2 Effects of hydrothermal changes on soil salinity and weed growth

Under nonmulching cultivation, strong surface evaporation and severe surface salt aggregation occur during the cotton reproductive period (especially the early reproductive period), which discourages cotton seed germination and seedling growth (Munawar et al., 2021; Wang et al., 2022a). Research on intercropping cotton with *Suaeda salsa* has shown that intercropping can effectively reduce soil salinity and improve soil water productivity and cottonseed yield (Liang and Shi, 2021). This study revealed that in the 0–15-cm soil layer during the entire growth period, compared with the CK treatment, ID1 and ID2 reduced the soil salinity by 1.0–38.8% and 6.0–35.2%, respectively. However, in the 15–45 cm soil layer during the symbiosis period, the soil conductivity at point P1 followed the order of intercropping > monocropping and increased with increasing intercropping cumin density (**Figure 6**). This pattern may be due to the varying ground cover densities, which increase the soil temperatures and consequently reduce surface evaporation, preventing salt from returning from the soil surface to deeper soil layers (Bi et al., 2010). During the nonsymbiotic period, the conductivity was highest in the CK treatment and lowest in the ID1 treatment in the 30-45 cm soil layer. On the one hand, intercropped cotton enters a rapid growth phase, consumes more crop water, and absorbs more salt in the soil (Liang and Shi, 2021). On the other hand, subsurface irrigation after cumin harvesting transports salts upward, and the damage caused by salt to roots in deep soil layers is reduced (Chen et al., 2024).

Planting cover crops is an effective weed suppression method that inhibits weed growth and aids in reducing pesticide use by occupying the ecological niches of weeds and directly competing with weeds for resources such as light, water, and nutrients during the early stages of crop growth (Pouryousef et al., 2015; Jha et al., 2017). Previous studies have shown that the weed suppression effect increases with increasing cover crop planting density (Majid et al., 2022), which is consistent with the results of this study. However, excessive intercropping can increase the degree of competition between cover crops and main crops, which is detrimental to yield. The period of 4–8 weeks after cotton emergence is critical for competition between weeds and cotton, and the effectiveness of weed control during this period is directly related to crop yield (Manalil et al., 2017). In this study, the bud stage (4–6 weeks after seedling emergence) was associated with cumin growth (Zhang et al., 2021) and high water requirements (**Table 2**). With increasing density of intercropped cumin, the soil water content significantly decreased (**Figure 3**), whereas the weed strain control effect and fresh weight control effect gradually increased with increasing weed inhibition (**Table 3**).

### 4.3 Effects of soil microenvironmental changes on cotton yield and the land equivalent ratio

Selecting reasonable intercropping ratios and density collocation points facilitates crop vegetative organ biomass accumulation and an appropriate distribution to improve crop economic yields (Zhang et al., 2020). The cotton yield is closely related to factors such as soil water, temperature, salt levels and air quality (Wang et al., 2021; Wu et al., 2022a). In this study, during the cotton/cumin intercropping symbiosis period, the land equivalent ratio was significantly negatively correlated with the electrical conductivity in the 0–15 cm soil layer, the soil water content in the 30–45 cm soil layer, the soil respiration rate, and weed density. These findings indicate that during the intercropping symbiosis period, deep soil moisture is fully utilized, effectively reducing soil surface salinity and suppressing soil carbon emission and weed growth. In addition, previous studies showed that when jujube trees are intercropped with cotton, a higher cotton planting density corresponds to more bolls and a greater yield. However, this study revealed that ID3 had fewer bolls and a lower boll weight than did CK in terms of seed cotton yield (**Table 4**). This decrease may be due to increased competition for water and nutrients from intercropped high-density cumin (Zhang et al., 2020), which significantly delays the early development of cotton (Du et al., 2016), shortens the boll opening stage and decreases the total number of bolls. In addition, there was no significant difference in seed cotton yield among ID1, ID2 and CK. ID1 and ID2 promoted the use of deep water by nonmulching crops by maintaining a high soil temperature and deep water consumption during the symbiotic period, which increased the crop yield (**Table 4**). The crop yield with intercropping increases with water consumption and soil temperature (Wu et al., 2022a; Ai et al., 2021). These findings indicate that a suitable cotton/cumin intercropping density does not significantly reduce the yield of seed cotton.

**Table 4:** Effects of cotton/cumin intercropping on crop yield and the land equivalent ratio.

Year	Treatments		Seed yield (kg ha^-1^)	Total number of bolls (×10^4^ ha^-1^)	Boll weight (g)	Intercropping cumin yield (kg ha^-1^)	WUE	LER	Am
2022	CK		5813.62a	115.13ab	5.04ab	——	——	——	——
	ID1		5921.47a	120.68a	4.90b	237.88c	——	1.63a	0.41a
	ID2		5859.62a	105.42b	5.23a	426.24a	——	1.70a	0.22b
	ID3		5449.08a	109.58ab	4.98b	305.16b	——	1.59a	0.28ab
2023	CK		5716.70ab	111.08ab	5.24a	——	5. 05b	——	——
	ID1		6027.50a	117.46a	5.14a	364.88b	5.45a	1.71a	0.39a
	ID2		5902.27ab	116.01ab	5.08a	548.44a	5.34ab	1.72a	0.34a
	ID3		5677.02b	113.16ab	4.86a	491.17a	5.15ab	1.67a	0.32a
P value									
Treatment	*		NS	NS	**	——	NS	NS
Year	NS		NS	NS	**	——	NS	NS
Treatment × year	NS		NS	NS	NS	——	NS	NS

WUE, water use efficiency; LER, land equivalent ratio; Am, aggressivity. Means within a column followed by a different letter are significantly different (P < 0.05) according to Duncan’s multiple range test. *p<0.05, **p<0.01, ns: *p*>0.05.

Interspecific interactions and competition affect crop growth and development; however, the factors that influence interspecific relationships include the crop mix, spatial distribution of plants, and environmental factors (Yin et al., 2020). Notably, the proportion of planted intercrops, the spacing between rows and the length of the symbiotic period affect the crop yield (Hailu, 2015). This study revealed that there was no significant difference in LER among the intercropping treatments (**Table 4**), and ID2 had a significantly greater Am than did ID1 in 2022. According to the “competition-recovery production principle”, early-harvest crops potentially support the growth of late-harvest crops (Zhang et al., 2021), but an excessive planting density intensifies intraspecific competition, which is not conducive to crop growth (Zhang et al., 2020). In this study, although the seed cotton yields of the ID1 and ID2 treatments were 3.5% and 4.5% lower than the average yield of mulched cotton in Xinjiang (National Bureau of Statistics of China, 2022), the nonfilmed intercropping planting pattern increased the land output while reducing the use of drip irrigation materials, water, and fertilizer and decreasing the labor requirement. Compared with the CK treatment, the ID2 treatment had more obvious resource utilization and yield advantages, which comprehensively improved the economic benefits of nonfilmed cotton fields and compensated for the economic and yield losses caused by not covering the crop with film.

Under conditions of nonfilm cultivation, planting early-maturing cotton plants can shorten the growth period and promote early boll opening. However, intercropped cumin exacerbates the lag in the cotton reproductive period and shortens the cotton boll opening time, which affects yield. Future research on intercropping productivity should focus on the suppression of crop growth by interspecific competition during the symbiotic period and on the water and fertilizer management of late-maturing crops to ensure that the intercropping of cotton and cumin results in an optimized yield and efficiency under no-film conditions.

## 5 Conclusions

The combination of nonfilmed deep drip irrigation and cotton/cumin intercropping can exploit the interspecific advantages of hydrothermal resources through temporal and spatial stratification, suppress salt accumulation and weed growth, and improve crop productivity. Compared with monocropping, intercropping with dense cumin (ID2) significantly increased crop water consumption and bare surface temperatures; ID2 also reduced soil electrical conductivity, soil carbon emissions, and weed density by increasing crop surface coverage. The ID1 treatment had the highest seed yield and aggressivity, whereas the ID2 treatment had the highest land equivalent ratio, and ID2 did not significantly differ from ID1 in terms of seed cotton yield. Therefore, to ensure crop yield and economic benefits, when the intercropping density of cumin reaches 8×10^5^ plants ha^-1^, to ensure that the cotton production is not reduced, water, heat and land resources are fully utilized to obtain greater economic benefits. The results of this study are highly practical for facilitating efficient resource utilization and environmental protection in the cotton industry in the future.

## Data Availability

The original contributions presented in the study are included in the article/Supplementary Material. Further inquiries can be directed to the corresponding authors.
